# Valvulopathies and Genetics: Where are We?

**DOI:** 10.31083/j.rcm2502040

**Published:** 2024-01-29

**Authors:** Mònica Coll, Anna Fernández-Falgueras, Anna Iglesias, Ramon Brugada

**Affiliations:** ^1^Unitat de Genòmica i Medicina Personalitzada, Laboratori Clínic Territorial, Institut Català de la Salut, 17003 Salt, Spain; ^2^Cardiovascular Genetics Center, University of Girona-Institut d'Investigacions Biomèdiques de Girona (IDIBGI), 17003 Salt, Spain; ^3^Cardiology Service, Hospital Josep Trueta, University of Girona, 17004 Girona, Spain; ^4^Centro de Investigación Biomédica en Red de Enfermedades Cardiovasculares (CIBERCV), 28014 Madrid, Spain; ^5^Medical Science Department, School of Medicine, University of Girona, 17004 Girona, Spain

**Keywords:** mitral valve prolapse, bicuspid aortic valve, non-syndromic forms, syndromic forms, hereditary, connective disorders

## Abstract

Valvulopathies are among the most common cardiovascular diseases, significantly increasing morbidity and mortality. While many valvular heart diseases are acquired later in life, an important genetic component has been described, particularly in mitral valve prolapse and bicuspid aortic valve. These conditions can arise secondary to genetic syndromes such as Marfan disease (associated with mitral valve prolapse) or Turner syndrome (linked to the bicuspid aortic valve) or may manifest in a non-syndromic form. When cardiac valve disease is the primary cause, it can appear in a familial clustering or sporadically, with a clear genetic component. The identification of new genes, regulatory elements, post-transcriptional modifications, and molecular pathways is crucial to identify at-risk familial carriers and for developing novel therapeutic strategies. In the present review we will discuss the numerous genetic contributors of heart valve diseases.

## 1. Introduction

The heart utilizes four valves to manage both pulmonary and systemic blood flow effectively. The mitral and tricuspid valves, known as atrioventricular valves, act as dividers between the atria and ventricles. They open during diastole, permitting the ventricles to fill with blood. Conversely, the aortic and pulmonary valves, collectively referred to as semilunar valves, open during ventricular systole, allowing blood to flow into pulmonary and systemic circulation. Valvular heart disease encompasses two primary conditions: stenosis, a narrowing of the valve that reduces blood flow, and regurgitation, the flow of blood back into the previous chamber. These valve-related issues can either be present from birth (congenital) or acquired during life. Although the heart can tolerate a certain degree of stenosis and regurgitation, if these conditions become severe, they can lead to heart failure.

Valvular heart diseases incidence vary significantly worldwide. High-income countries tend to experience a higher prevalence of functional and degenerative valvular diseases, whereas low-income and middle-income countries predominantly contend with rheumatic heart disease. This distribution pattern is mirrored by the fact that rheumatic heart disease remains the most prevalent form of valvular heart disease worldwide, affecting approximately 41 million individuals [1]. In the developed world, the most common valve disorder affecting the heart is aortic valve stenotic disease, responsible for 61% of all valvular heart disease-related deaths [2]. Diseases affecting the mitral valve make up 15% of these cases, and they often have a significant genetic influence [2].

Valvular heart diseases are mostly acquired during adult life, however, familial clustering and heritability of common valve disorders such as mitral valve prolapse, or bicuspid aortic valve highlight the significant role of genetic factors. Identification of these genes is crucial to the management of at-risk family members as well as improving therapeutic strategies. Although international guidelines recommend family screenings, these are not uniformly conducted and can be sporadic and unevenly distributed [3, 4, 5]. A recent study conducted by Bray *et al*. [6] examined 6,054 family members of individuals diagnosed with BAV (Bicuspid Aortic Valve), and found the prevalence of BAV among these relatives was 7.3%. However, in some screened families, the prevalence of BAV reached as high as 23.6% [6].

**Fig. 1.**
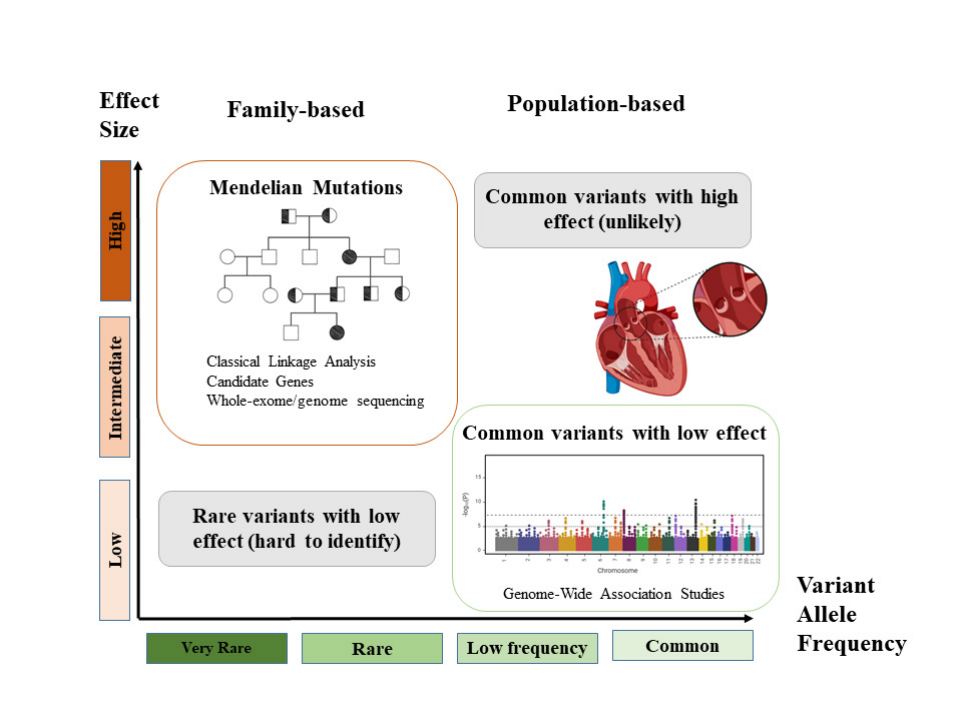
**Genetic variants and human diseases**. Variants with a high effect size are normally very rare and follow a Mendelian pattern of inheritance. In contrast, common variants in the general population have lower effects in the disease. Rare variants with low effect or common variants with high effect do not occur frequently.

Traditionally, large families with well-documented histories have been crucial in determining the genetic basis of diseases thought genome-wide linkage analysis. However, with the advent of high-throughput technologies such as whole-exome and whole genome sequencing, as well as array comparative genome hybridization, the identification of new loci, associated genes, and chromosomal rearrangement has been accelerated. *In vivo* and *in vitro *functional studies have been decisive in dissecting molecular pathways. Nevertheless, the genetic research into valvulopathies is complicated by factors such as incomplete penetrance, phenotypic heterogeneity, and possible modifier factors which can obscure the genetic landscape of these diseases (Fig. 1).

## 2. Mitral Valve

**Fig. 2.**
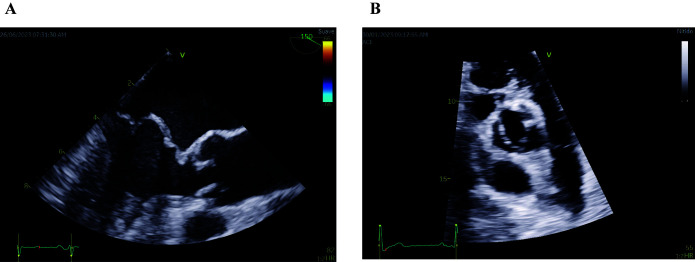
**Echocardiographic images of mitral valve prolapse of both leaflets (A) and bicuspid aortic valve (B)**. (A) Midesophageal long-axis transesophageal echocardiographic image depicting mitral valve prolapse of both leaflets. Note that both leaflets are beyond the valvular plane, protruding into the left atrium, when the mitral valve is closed (B) Parasternal short-axis transthoracic echocardiographic image showing a bicuspid aortic valve with fusion of both coronary leaflets. A raphe is present between the fused leaflets.

Mitral valve regurgitation (MR) is caused by defects in the mitral leaflets, chordae or papillary muscles. The primary cause of MR is mitral valve prolapse (MVP) [7]. The most common valvular heart disease observed in Western countries, MVP, affects 2–3% of the general population and is defined as systolic displacement of one or both mitral leaflets ≥2 mm above the plane of the mitral annulus in the sagittal view of the mitral valve (Fig. 2A). The prevalence of MVP varies with age, tending to increase as individuals get older. Sex differences are also present, with a higher MVP prevalence among females [8]. However, the rates of MVP remain consistent across different ethnic groups.

The MVP condition is generally considered benighm in the absence of MR and left-ventricular abnormalities. However, there is a small and poorly defined group of individuals who are at elevated risk for dangerous ventricular arrhythmias and sudden cardiac death (SCD) due to MVP. While MVP-related SCD occurs in less than 1% of all MVP cases annually, this incidence rises sharply to 4%–7% among younger individuals experiencing SCD with MVP. To pinpoint those with arrhythmic MVP, clinical assessments, echocardiography, and magnetic resonance imaging prove to be useful diagnostic tools [9].

There are two main types of MVP, myxomatous MVS or Barlow’s disease and fibroelastic deficiency (FED). Myxomatous MVP is characterized by an abundance of valve tissue, including thickened or elongated chordae (the tendinous strings that support the valve leaflets), dilation of the valve annulus (the ring-shaped structure to which the valve is attached), and sometimes calcification. In this type of MVP, the likelihood of chordal rupture is relatively low and MVP occurs more frequently in the elderly. FED is the most common form of MVP occurring earlier in life and it is characterized by chordal thinning, elongation and/or high probability of rupture, with classic findings of prolapse and MR of variable severity.

In the past, the clinical diagnosis of MVP was based on the presence of different signs and symptoms such as dyspnea, chest pain, and electrocardiographic abnormalities. Risk stratification of patients with MVP includes focused history, 12-lead electrocardiogram (ECG), extended ECG monitoring and detailed echocardiography [10]. MVP can be part of a well-defined syndrome (secondary cause) or can be identified in a non-syndromic context (primary cause). In non-syndromic cases, mitral prolapse may appear sporadically (non-familiar) or in familiar circumstances (hereditary).

### 2.1 Primary or Non-Syndromic Forms of Mitral Valve Prolapse

Non-syndromic sporadic forms are the most common conditions of MVP. As with other common diseases, these cases are often thought to result from a combination of common genetic variants with weak addative effects that lead to a cumulative pathophysiological impact. Identification of the genetic background in non-syndromic MVP cases has been possible with the use of high-throughput genomic technologies, with genome-wide association studies (GWAS) approach. Since the first GWAS performed by Dina *et al*. [11], several genes have been associated with sporadic MVP cases: *LIM and cysteine-rich domains protein 1 (LMCD1)*, *tensin-1 (TNS1)*, *glioma-associated oncogene homolog -similar 1 (GLIS1)*, *synaptotagmin 2 (SYT2)*, *methionine sulfoxide reductase A* (*MSRA)*, F-box protein 46 (*FBXO46)*, *spectrin β chain, non-erythrocytic 1 (SPTBN1)*, *latent transforming growth factor β-binding protein 2 (LTBP2), transforming growth factor β 2 (TGFB2), neuromedin B NMB*, and α*-protein kinase 3 (ALPK3) * [12, 13]. These associations highlight the genetic complexity and the polygenic nature of MVP.

In a considerable number of familial cases with a Mendelian inheritance pattern, researchers have uncovered non-syndromic cases of MVP where certain rare genetic variations exert a profound effect on the development of the condition. Autosomal dominant pattern of inheritance is most common, although X-linked patterns have also been identified. As in other hereditary diseases, the first approach to identify candidate regions was through classical linkage analysis. These studies allowed for the first time the identification of the regions Xq28, 16p11.2-p12.1 (*MMVP1*), 11p15.4 (*MMVP2*) and 13q31.3-31.2 (*MMVP3*) as candidate regions to explain these hereditary cases. Advances in next generation sequencing technologies, such as whole exome and whole genome sequencing, continue to refine our understanding by identifying new candidate genes associated with familial MVP.

Until recently, only three genes have been identified in familial cases, with most of them exhibiting genetic segregation. However, there has been significant expansion in this area lately. In the 60s, *Monteleone* and *Fagan* [14] introduced the first evidence of X-linked inheritance in MVP cases, while researchers from the early 2000s focused on the chrXq28 [14, 15, 16]. In 2007 the *filamin A (FLNA)* gene was discovered and given the name Filamin A-MVP. *FLNA* is located on the chr Xq28 and the X-linkage pattern of inheritance causes full penetrance in men and incomplete in women [17]. In 2019, Bains *et al*. [18] identified a truncating mutation in *FLNC* gene (sarcomeric protein filamin C) in a case of arrhythmogenic leaflets forms of MVP that showed segregation. Mutations of the *FLNC* gene have been definitively associated with hereditary cardiomyopathies such as dilated cardiomyopathy, hypertrophic cardiomyopathy and restrictive cardiomyopathy. In 2015, Durst *et al*. [19] identified the second associated gene with MVP, *DCHS1* (Dachsous 1). A loss-of-function variant showed segregation in a large family with myxomatous MVP and posterior functional *in-vivo* studies confirmed this association. It was not until 2019 when the third gene, *DAZ-interacting protein 1 (DIZIP1)*, was identified by Toomer *et al*. [20] in a large family of MVP and the pathogenesis was confirmed in a mouse model. Although several studies support a genetic background for the non-syndromic MVP, the recent consensus published by the European Society of Cardiology does not consider the routine genetic testing for these cases [10].

### 2.2 Secondary or Syndromic Forms of Mitral Valve Prolapse

MVP can also occur as a secondary condition associated with syndromic disorders, which are often inherited connective tissue diseases. In such cases, researchers have identified mutations in various genes that encode structural proteins, modifying enzymes, or components of transforming growth factor-β (TGFβ)-signaling pathway as contributing factors to the development of MVP. Syndromes with a high prevalence of MVP are Marfan syndrome (MFS), Mitral Aorta Skeleton and Skin Phenotype and Loeys-Dietz syndrome. These syndromes exhibit certain similarities in their manifestation, with overlapping features involving the cardiovascular system, skeletal structures, and the skin (Table 1, Ref. [11, 12, 13, 21, 22, 23, 24, 25, 26, 27, 28, 29, 30, 31]).

**Table 1.** **Genetic causes of mitral valve prolapse**.

**Syndromic MVP**	**Associated genes**	**Prevalence of MVP**	**Reference**
Marfan Syndrome	*FBN1* (>90% cases); *FBN2, TGFBR1, TGFBR2, LTBPs 1-3, SKI*	40–68%	[21]
Mitral Aorta Skeleton and Skin Phenotype (MASS)	*FBN1*	75%	[22]
Loeys-Dietz syndrome	*TGFBR1* (20–23%), *TGFBR2* (55–60%), *SMAD2* (1–5%), *SMAD3* (5–10%), *TGFB2* (5–10%), *TGBF3* (1–5%)	21% type 5	[22]
		17% type 4	
		5–13% types 1, 2 and 3	
Pseudoxanthoma elasticum	*ABCC6*	43.4%	[26, 27]
Familial Myxomatous Valvular Degeneration	*FLNA*	38.1%	[22, 29]
Fragile-X syndrome	*FMR1* (>200 CGG repeats)	37.8%	[22]
Aneuploidy	Trisomy 18, 13, 15, 21	31.4%	[28]
Ehler-Danlos (Cardiac-valvular form)	*COL1A2*	6.2%	[23]
Adult polycystic kidney disease	*PKD1, PKD2*	21.4%	[22]
Ebstein anomaly	*MYH7, NKX2, filamin A, GATA4, *channel genes such as the V-type voltage-gated sodium channel amb *TPM1*	11.4%	[24, 30]
Osteogenesis imperfecta	*COL1A1, COL1A2* (85–90%)	5.4%	[25]
Juvenile polyposis syndrome	*SMAD4*	-	[31]
**Non-Syndromic MVP and familial forms**	*FLNA, DCHS1, DZIP1, FLNC*	-	
**Non-Syndromic MVP and sporadic forms**	*LMCD1, TNS1, GLIS1, SYT2, MSRA, FBXO46, SPTNB1, LTBP2, TGFB2, NMB, ALPK2*	-	[11, 12, 13]

MVP, mitral Valve Prolapse; *FBN1*, fibrillin-1; *FBN2*, fibrillin-2; *TGFBR1*, transforming growth factor β receptor 1; *TGFBR2*, transforming growth factor β receptor 2; *LTBPs*, latent transforming growth factor-binding proteins (1-3); *SKI*, ski proto-oncogene; *SMAD2*, SMAD (mothers against decapentaplegic) homolog 2; *SMAD3*, SMAD (mothers against decapentaplegic) homolog 3; *TGFB2*, transforming growth factor β 2; *TGFB3*, transforming growth factor β 3; *ABCC6*, ATP binding cassette subfamily c member 6; *FLNA*, filamin A; *FMR1*, fragile X mental retardation 1; *COL1A2*, collagen type I α 2 chain; *PKD1*, polycystic kidney disease 1; *PKD2*, polycystic kidney disease 2; *MYH7*, myosin heavy chain 7; *NKX2*, NK2 Homeobox (specifically, NKX2-5); *TPM1*, tropomyosin 1; *COL1A1*, collagen type I α 1 chain; *SMAD4*, SMAD (mothers against decapentaplegic) homolog 4; *DCHS1*, dachsous cadherin-related 1; *DZIP1*, DAZ-interacting zinc finger protein 1; *FLNC*, filamin C; *LMCD1*, LIM and cysteine-rich domains protein 1; *TNS1*, tensin-1; *GLIS1*, glioma-associated oncogene homolog-similar 1; *SYT2*, synaptotagmin 2; *MSRA*, methionine sulfoxide reductase A; *FBXO46*, F-box protein 46; *SPTNB1*, spectrin beta chain, non-erythrocytic 1; *LTBP2*, latent transforming growth factor beta-binding protein 2; *NMB*, neuromedin B; *ALPK2*, α-protein kinase 2; *GATA4*, GATA binding protein 4.

#### 2.2.1 Marfan Syndrome

As an autosomal dominant disease MFS is an age-related genetic disorder of the connective tissue with prominent manifestations in the skeletal, ocular, and cardiovascular systems, and has an estimated incidence of 1 in 5000 individuals [32]. In patients with MFS, both MVP and MVR are well-established complications. The prevalence of MVP in adults with MFS is much higher (40–68%) than in the general population (1–2%) [33]. *Fibrillin-1* (*FBN1*) gene-mutations account for about 90% of MFS cases and up to 25% of cases are due to *de novo *variants, variants identified for the first time in an individual and not inherited from a parent because of a mutation in a germ cell. Fibrillin-1 plays a crucial role as the primary component of extracellular microfibrils, which provide essential support to connective tissues, especially in arteries, pericondrium (the connective tissue surrounding cartilage), and various structures within the eye. Although there is a very clear gene-phenotype association, other genes such as *FBN2*, *transforming growth factor β receptor 1 (TGFBR1)*, *transforming growth factor β receptor 2 (TGFBR2)*, *latent transforming growth factor β -binding protein 1 (LTBP-1)*, *latent transforming growth factor β -binding protein 2 (LTBP-2)*, *latent transforming growth factor β -binding protein 3 (LTBP-3)* and *ski proto-oncogene (SKI)* have been described in MFS cases [21].

#### 2.2.2 Mitral Aorta Skeleton and Skin Phenotype (MASS)

The MASS phenotype is a condition with marfanoid characteristics that doesn’t meet the Ghent criteria for the diagnosis of MFS. The prevalence of MVP in MASS phenotype is around 75% and although the genetic cause is still unknown, some of them show a *FBN1* mutation [22]. This suggests a potential genetic overlap between the MASS phenotype and other connective tissue disorders.

#### 2.2.3 Loyes-Dietz Syndrome

Loeys-Dietz syndrome (LDS) is a rare disorder characterized by an autosomal dominant mode of inheritance, and it was initially identified and described in 2005 by Bart Loeys and Harry Diet [34]. In comparison to MFS, individuals with LDS tend to exhibit more severe cardiovascular manifestations. Aortic aneurysms in LDS have a higher likelihood of dissection or rupture at smaller diameters and at a younger age compared to those seen in MFS patients.

Prolapse and insufficiency or mitral valve are more frequent in LDS than in general population (more common in LDS types 4 [17%] and 5 [21%] than types 1, 2 and 3 [5–13%]) but in less extent than in Marfan. The prevalence of MVP in LDS is close to 25%, seeming counterintuitive that a more aggressive marfanoid syndrome would cause a lower prevalence of MVP [22]. In LDS, components of the TGFβ signaling pathway are altered, including the actual cytokines (TGFB2/3), the receptors (TGFBR1/2), and the downstream effectors (SMAD2/3). As in MFS, *de novo *mutations often occur in LDS cases.

#### 2.2.4 Other Syndromics Forms Causing MVP

While MVP has been linked to various other syndromic conditions, currently there is insufficient data to determine the exact prevalence of MVP in these cases. One such condition is Ehlers-Danlos Syndrome (EDS), which encompasses a diverse group of connective tissue disorders sharing similar clinical features. These features include joint hypermobility, tissue fragility, and skin hyperextensibility [23]. Up to 13 subtypes of EDS have been described, with the cardiac-valvular —specifically linked to recessive loss-of-function mutations in *collagen type I α 2 chain* (*COL1A2)*—displaying heart-related issues [23]. The estimated prevalence of MVP in this EDS subtype appears relatively low at 6.2%, and its association with EDS is debated [35, 36]. As in other connective tissue disorders, osteogenesis imperfecta has also been associated with a slightly increase prevalence of MVP (5.4%) [24]. About 85–90% of cases are associated with dominantly-inherited pathogenic variants of *collagen type I α 1 chain* (*COL1A1)* or *COL1A2 * [25]. Pseudoxanthoma elasticum, characterized by the calcification of elastic fibers within arteries, eyes, and skin is caused by biallelic pathogenic variants in the *ATP binding cassette subfamily c member 6 (ABCC6)* gene, and results in an increased prevalence of MVP at 31.4%, which is notably higher than the general population [26, 27, 37].

Williams-Beuren syndrome is an infrequent autosomal dominant disorder characterized by a combination of cardiovascular, connective tissue, and central nervous system abnormalities, along with mild intellectual disability and a sociable and outgoing personality. Among the cardiovascular abnormalities in this condition, MVP is commonly reported. Across ten studies, the median prevalence of MVP in Williams-Beuren syndrome is approximately 22.3%. The syndrome is caused by a 1.5Mb deletion in chromosome 7q11.23, which encompasses around 26–28 genes, including the elastin gene [22].

Aneuploidy syndromes, which can include a trisomy of 18, 13, 15, or 21, involve the presence of abnormal numbers of chromosomes, and are associated with various cardiac malformations, particularly alterations to heart valves. The prevalence of MVP is also elevated in Down Syndrome, with a median prevalence of 31.4% across seven studies, as described in a recent review [22, 38]. While the pathophysiology is still unknown, chromosome 21 is known to code for two subunits of collagen VI [28].

Familial Myxomatous Valvular Degeneration (FMVD) refers to a group of disorders with a diverse range of characteristics, all sharing the common feature of myxomatous degeneration affecting multiple heart valves [39]. It is also known by the term “familial cardiac valvular dystrophy” . The median prevalence of MVP in patients with FMVD is 38.1%. Mutations to the *filamin A* (*FLNA)* gene have been associated with this disorder [39]. *In vivo *experiments eliminating *FLNA* have shown that it leads to the enlargement of the mitral valve during the fetal stage. As these organisms grow, this condition progresses to MVP by the time they reach 2 months of age [29].

Another common hereditary syndrome is adult polycystic kidney disease (APKD). Prevalence of MVP in APKD is estimated 21.4% and the main genes associated are *polycystic kidney disease 1 (PKD1)* and *polycystic kidney disease 2 (PKD2) * [22]. Mutations that affect the genes involved in primary cilia function can hinder the protein’s proper localization to these structures, leading to cyst development. In 2019, *DAZ-interacting zinc finger protein 1 (DZIP1)* was identified as a causative gene for idiopathic non-syndromic MVP, expanding our understanding of the genetic underpinnings of this condition [20].

Ebstein anomaly is a congenital heart defect primarily affecting the tricuspid valve. There are limited reports of cases with malformation of the mitral valve, with a prevalence of approximately 11.4% [22]. Comprehensive genetic studies on Ebstein’s anomaly are scarce, but some research suggests associations with specific genes such as *myosin heavy chain 7* (*MYH7)*, *NK2 homeobox 5 *(*NKX2-5)*, *FLNA*, *GATA binding protein 4 (GATA4)*, channel genes like the *V-type voltage-gated sodium channel (SCN5A)*, and *tropomyosin 1 (TPM1) * [30].

Fragile X syndrome is a hereditary condition characterized by intellectual disability and is attributed to a trinucleotide repeat disorder [40]. It occurs when there is an expansion of CGG repeats in the *fragile X mental retardation 1 (FMR1)* gene to a number greater than 200, which results in the gene becoming silenced. Pathophysiological mechanisms for MVP in fragile X syndrome are still unknown, however, data from six publications estimate the prevalence of MVP in 37.8%, which is likely overestimated [22].

The link between juvenile polyposis syndrome (JPS) and its association with MVP has been documented in a single publication involving a family with a history of aortopathy, mitral valve dysfunction, and JPS [31]. In this family, a nonsense mutation in the *SMAD4* gene was identified. The connection between these conditions is not unexpected, as both *SMAD4* and TGFβ are known to interact within the same signaling pathway [23, 31].

Mitral valve stenosis is generally caused by rheumatic heart disease and nearly 25% of cases with this condition will suffer valve affectation [41].

## 3. Tricuspid Valve

Tricuspid valve stenosis is an uncommon condition primarily caused by rheumatic heart disease and less commonly due to secondary to factors like tumors [42]. Alternatively, tricuspid valve regurgitation (TVR) can have different underlying causes [42]. Primary TVR might be congenital, either as an isolated defect or associated with atrioventricular canal defects, ventricular septal aneurysms, or as a part of Ebstein’s anomaly [43]. Primary TVR may also be of congenital origin, either as an isolated lesion or in association with atrioventricular canal defects, aneurysms of the ventricular septum or as a component of Ebstein’s anomaly. This rare defect affecting between 0.39–0.72 cases per 10,000 births is characterized by downward displacement of the tricuspid valve into the right ventricle [44].

The *MYH7 *has been identified as the strongest genetic link to both sporadic and familial cases of this disease, however associations with other genes such as *TPM1, Kelch like family member 26 (KLHL26), FLNA *or *NKX2-5* are also related to Ebstein’s anomaly, albeit with less supporting data [30, 45, 46, 47]. Additionally, TVR can be an acquired condition, commonly due to infective endocarditis.

Secondary TVR can be seen in individuals with right ventricular pressure overload resulting from various cardiac or pulmonary vascular conditions [42]. The most prevalent causes are left-sided valvular diseases that lead to an increase in the volume and pressure on the right side of the heart.

## 4. Aortic Valve

The aortic valve is responsible for regulating the flow of blood from the left ventricle of the heart into the aorta, which is the primary artery responsible for carrying oxygen-rich blood to the entire body. Congenital malformations are the main cause of aortic valve stenosis; however, calcification or rheumatic disease can also be responsible.

BAV is the prevailing valve abnormality found in around 0.5–2% of the general population [48]. It is characterized by an aortic valve that has only two cusps, or flaps, instead of the usual three (Fig. 2B). BAV follows an autosomal pattern of inheritance with incomplete penetrance, variable expressivity and male predominance in a 3:1 ratio [49]. More than 35% of individuals with BAV will experience various complications, including aortic valve stenosis and regurgitation, as well as ascending aortic aneurysms and dissection. Studies have shown a high incidence of thoracic aortic aneurysms (TAA) in BAV patients and their family members, suggesting that both TAA and BAV may share a common genetic cause [50, 51].

As in other valvulopathies, BAV can also be grouped as sporadic BAV (sporadic isolated defect), familial non-syndromic BAV (identified in clusters within families without associated anomaly), or syndromic BAV (considered familial and associated with other anomalies including cardiovascular defects) [52]. It is well established that BAV has a strong genetic component as many studies have shown familial clustering of BAV.

Genetic factors play a significant role in the development of BAV, with observed heritability ranging from 47% to 89% [48]. This broad range reflects the diverse genetic nature of BAV, which encompasses a wide array of disease-causing mutations and risk variants. There are both monogenic forms of BAV, which can be syndromic or non-syndromic. Syndromic BAV includes conditions like Loeys-Dietz syndrome, associated with mutations in *TGFBR1* or *TGFBR2*, and familial thoracic aortic aneurysm and dissection (TAAD) syndrome, linked to mutations in actin, α 2, smooth muscle, aorta (*ACTA2)*, presenting with additional extra-cardiac manifestations [53]. However, the majority of BAV cases are sporadic, arising from a complex interplay of genetic factors.

### 4.1 Primary or Non-Syndromic Forms of Bicuspid Aortic Valve

While it is well established that BAV has a heritable component, the precise genetic factors underlying BAV and its related conditions are still not fully understood. As of now, *notch receptor 1 (NOTCH1)* is the only confirmed candidate gene that has been linked to both familial and sporadic cases of BAV [54]. Despite ongoing research, there is still much to be discovered about the genetic basis of BAV and its associated diseases.

The first association was in 2005 by Garg *et al*. [54], when they associated mutations in *NOTCH1* gene with non-syndromic BAV. Other genes such as *GATA4, GATA binding protein 5 (GATA5), GATA binding protein 6 (GATA6), SMAD (mothers against decapentaplegic) homolog 6 (SMAD6), roundabout guidance receptor 4 (ROBO4), methionine adenosyltransferase 2A (MAT2A), a disintegrin and metalloproteinase with thrombospondin motifs 19 (ADAMTS19), NKX2–5, T-box 20 (TBX20), FBN1* have also been associated with non-syndromic BAV with different degrees of supporting evidence [55]. The GATA genes (*GATA4*, *GATA5*, and *GATA6*) are responsible for encoding zinc finger transcription factors that play a crucial role in regulating the early expression of genes related to cardiac development and differentiation of cardiac cell lineages [56]. Rare variations in any of the GATA genes have been linked to familial BAV with a pattern of autosomal inheritance [57]. Additionally, Smad proteins serve as intracellular mediators of signal transduction for transforming growth factor-beta (TGF-β) and bone morphogenetic protein ligands. In cases of non-syndromic BAV and TAA, specific rare missense and loss-of-function variants in *SMAD4* and *SMAD6* have been identified [57]. These findings highlight the role of these genetic factors in the development of BAV and associated conditions [58]. Although familial genetic analysis supported by *in vivo* studies suggested *ROBO4* as a new candidate gene to explain BAV and TAA, a more recent study indicates that cohorts with BAV are enriched with variants in these genes, however they do not significantly contribute to BAV in cohorts with TAA, concluding that BAV patients who present with TAA are a genetically distinct subgroup with implications for genetic testing and prognosis [55, 59]. Finally, although *FBN1* is typically associated with MFS, common and rare variants have been identified in cases of non-syndromic BAV or in BAV patients with aortic root aneurysms but without features of MFS [60, 61].

Around 20% of non-syndromic hereditary TAADs are caused by changes in the contractile apparatus of vascular smooth muscle cells, influenced by specific genes such as *ACTA2*, *myosin heavy chain 11 (MYH11)*, *myosin light chain kinase (MYLK)*, and *protein kinase, CGMP-dependent, type I (PRKG1)* [62]. However, the genetic basis for the remaining 80% of familial TAAD cases remains elusive as it cannot be accounted for by the candidate genes previously identified and studied. Further research is needed to unravel the underlying genetic factors responsible for these unexplained familial TAAD cases [62].

In patients with non-syndromic and sporadic cases of BAV, single gene mutations are implicated in less than 10% of cases, and are associated with a variety of molecular pathways [52]. This suggests a complex genetic landscape without a singular mechanism. To better comprehend the genetic basis of this group of patients, new high-throughput technologies have emerged, which not only analyze coding regions but also investigate non-coding regions or epigenetic modifications. These advanced techniques offer promising avenues to uncover the genetic background responsible for BAV cases with no identified single gene mutations and shed light on previously elusive genetic factors contributing to the condition.

### 4.2 Secondary or Syndromic Forms of Bicuspid Aortic Valve

BAV can manifest as part of a syndrome, appearing within a cluster of cardiac and noncardiac anomalies (Table 2, Ref. [55, 57, 62, 63, 64, 65, 66, 67]). The highest prevalence of BAV is found in Turner’s syndrome, resulting from partial or complete absence of one chromosome in females (45X). The high occurrence of BAV in Turner’s syndrome and the 3:1 male predominance may suggest the importance of the X-chromosome in BAV development. Furthermore, in 2018, Corbitt *et al*. [68] made a noteworthy discovery describing the co-occurrence of X chromosome *TIMP metallopeptidase inhibitor 1 (TIMP1)* hemizygosity and certain variants of its autosomal counterpart *TIMP metallopeptidase inhibitor 3 (TIMP3)* substantially elevate the risk of aortopathy in individuals with Turner syndrome.

**Table 2.** **Bicuspid aortic valve**.

**Syndromic BAV**	**Associated genes**	**Prevalence of BAV**	**Reference**
Turner Syndrome	Monosomy X or partial of X chromosome	15–40.7%	[66, 67]
Loeys-Dietz syndrome	*TGFBR2 *(55–60%), *TGFBR1* (20–25%),*TGFB2 *(5–10%), *SMAD3 *(5–10%), *TGFB3 *(1–5%)	10–30%	[57, 62]
Andersen syndrome	*KCNJ2*	10%	[63]
Shone’s complex	*NOTCH1*	50%	[57]
Velocardiofacial syndrome	deletion of 22q11.2	7.5%	[64]
**Non-Syndromic familial or sporadic forms of BAV**	*NOTCH1, GATA4, GATA5, GATA6, SMAD4, SMAD6, ROBO4, MAT2A, ADAMTS19, NKX2–5, TBX20, FBN1, ACTA2*	-	[55, 57, 65]
	*Polymorphisms *(*ACE*, *MMP*)		
**Familial TAAD**	*ACTA2 *(14–21%), *MYH11*, *MYLK,* and *PRKG1 *gene	-	[62]

BAV, bicuspide aortic valve; TAAD, thoracic aortic aneurysms and dissection; *TGFBR2*, transforming growth factor β receptor 2; *TGFBR1*, transforming growth factor β receptor 1; *TGFB2*, transforming growth factor β 2; *SMAD3*, SMAD family member 3; *TGFB3*, transforming growth factor β 3; *KCNJ2*, potassium voltage-gated channel subfamily J member 2; *NOTCH1*, NOTCH receptor 1; *GATA4*, GATA binding protein 4; *GATA5*, GATA binding protein 5; *GATA6*, GATA binding protein 6; *SMAD4*, SMAD family member 4; *SMAD6*, SMAD family member 6; *ROBO4*, roundabout guidance receptor 4; *MAT2A*, methionine adenosyltransferase 2A; *ADAMTS19*, a disintegrin and metalloproteinase with thrombospondin motifs 19; *NKX2-5*, NK2 homeobox 5; *TBX20*, T-box 20; *FBN1*, fibrillin 1; *ACTA2*, actin, α 2, smooth muscle, aorta; *ACE*, angiotensin I converting enzyme; *MMP*, matrix metallopeptidase; *MYH11*, myosin heavy chain 11; *MYLK*, myosin light chain kinase; *PRKG1*, protein kinase, CGMP-dependent, type I.

The BAV is associated with various syndromic conditions, each with distinct genetic underpinnings. Loeys-Dietz syndrome, which is present in approximately 10% of BAV cases, involves mutations in the transforming growth factor-β pathways genes (*TGFB2, TGFB3, TGFBR1, TGFBR2 *and *SMAD3*) [69]. Andersen syndrome is a very rare orphan genetic multisystem channelopathy without structural heart disease caused mainly by mutations in *potassium voltage-gated channel subfamily J member 2* (*KCNJ2)* gene [63]. However, it has been described a family with this disorder and BAV with a *KCNJ2* mutation that segregates within the family [63]. Shone’s complex includes multiple left heart obstructive lesions, and several reports include BAV as a common component of this syndrome [70]. While the genetic cause of this congenital disorder is still unknown, some studies indicate that recessive variants in *MYH6* could account for at least 11% of Shone’s cases [71]. Velocardiofacial syndrome (VCFS), the most common microdeletion syndrome in humans, manifests a wide variety of presentations and phenotypes including congenital heart disease resulting in BAV [64, 72]. VCFS is characterized by a deletion of 22q11.2 and, according to a report by Kobrynski and Sullivan, the specific *T-box transcription factor 1 (TBX1)* gene. However, at the locus of chromosome 22, the deletion site, there are more than 35 other genes, with many playing important roles in cardiac development including *histone cell cycle regulator* (*HIRA)*, *ubiquitin fusion degradation 1-like protein (UFD1L)*, *v-crk avian sarcoma virus CT10 oncogene homolog-like (CRKL)* and *DiGeorge syndrome critical region 6 (DGCR6)* [64, 72].

Although BAV was initially considered more common in MFS, recent studies demonstrated that the prevalence of BAV was 1.8%, which aligns with the prevalence of the general population [73]. Similar to other valvular diseases, a combination of multiple common genetic variants may act as modifier in the pathogenesis of BAV aortopathy, contributing to the variability of clinical phenotypes [65]. Aortic regurgitation can arise from abnormalities affecting either the aortic valve or the aortic root. The primary causes of this condition include post-inflammatory valve disease, infective endocarditis, congenitally bicuspid aortic valve, connective tissue diseases, and iatrogenic causes like valvuloplasty [74].

## 5. Pulmonary Valve

Pulmonary valve stenosis (PVS) is primarily a congenital condition and although acquired cases can also occur, are considerably less common [75]. The most common genetic contributor for PVS is Noonan syndrome (NS) (Table 3, Ref. [76, 77, 78, 79, 80, 81, 82]). This autosomal dominant congenital disorder, affecting 1:1000 to 1:2500 live births, is characterized by cardiac defects, with PVS and hypertrophic cardiomyopathy (HCM), and also involves multiple organ systems [83]. The primary cause of NS is attributed to gain-of-function mutations in genes that encode components or regulators of the RAS/mitogen-activated protein kinase (MAPK) signal transduction pathway. This condition is classified under the RASopathies family of disorders. The genes commonly involved in NS are *protein tyrosine phosphatase, non-receptor type 11 (PTPN11)* (found in approximately half of NS patients), along with others like *son of sevenless homolog 1 (SOS1)*, *RAF proto-oncogene serine/threonine-protein kinase (RAF1)*, *Kirsten rat sarcoma viral oncogene homolog (KRAS)*, *neuroblastoma RAS viral oncogene homolog (NRAS)*, *Ras-like without CAAX 1 (RIT1)*, which show moderate or limited association with the syndrome [76]. The causative mutations remain unidentified in 10%‒20% of patients, and *de novo* mutations account for most NS cases. Genetic testing, therefore, can help with risk assessment and patient management.

**Table 3.** **Pulmonary valve stenosis**.

**Syndromic PVS**	**Associated genes**	**Prevalence of PVS**	**Reference**
Noonan Syndrome	*PTPN11, SOS1, RAF1, KRAS, NRAS, RIT1 (definitive or strong)*	40%	[76]
	*MRAS, RRAS BRAF, SOS2, LZTR1, TASA2, RRAS, RRAS2, MAP2K1 (moderate or limited)*		
Tetralogy of Fallot	chromosomal abnormality: 22q11 microdeletions, trisomy 21, Alagille’s disorder, Cat Eye syndrome, or CHARGE and VATER/VACTERL syndromes	80%	[81, 82]
Williams syndrome (WS)	Deletion of chromosome 7 (7q11.23)	45.1%	[80]
**Non-Syndromic PVS**			
Tetralogy of Fallot	*NOTCH1, FLT4, NKX2.5, GATA4, GATA5, GATA6, TBX1, TBX2, TBX5, CITED2, ZFPM2/FOG2, FOXC1, FOXC2, FOXH1, HAND2, JAG1, FLNA, KDR, NDRG4, SMARCC2, RYR1, ZFPM1, CAMTA2, DLX6, PCM1, JAG1*	80%	[77, 78, 79]

PVS, pulmonary valve stenosis; *PTPN11*, protein tyrosine phosphatase, non-receptor type 11; *SOS1*, son of sevenless homolog 1; *RAF1*, RAF proto-oncogene serine/threonine-protein kinase; *KRAS*, Kirsten rat sarcoma viral oncogene homolog; *NRAS*, neuroblastoma RAS viral oncogene homolog; *RIT1*, Ras-like without CAAX 1; *MRAS*, Ras-related protein M-Ras; *RRAS*, Ras-related protein R-Ras; *BRAF*, v-Raf murine sarcoma viral oncogene homolog B; *SOS2*, Son of sevenless homolog 2; *LZTR1*, Leucine zipper transcription regulator 1; *TASA2*, TGF-beta stimulated clone 22; *RRAS2*, Ras-related protein R-Ras2; *MAP2K1*, Mitogen-activated protein kinase kinase 1; *NOTCH1*, Neurogenic locus notch homolog protein 1; *FLT4*, Fms-related tyrosine kinase 4; *NKX2.5*, NK2 homeobox 5; *GATA4*, GATA binding protein 4; *GATA5*, GATA binding protein 5; *GATA6*, GATA binding protein 6; *TBX1*, T-box transcription factor 1; *TBX2*, T-box transcription factor 2; *TBX5*, T-box transcription factor 5; *CITED2*, Cbp/p300-interacting transactivator 2; *ZFPM2/FOG2*, Zinc finger protein multitype 2 (also known as Friend of GATA 2); *FOXC1*, Forkhead box C1; *FOXC2*, Forkhead box C2; *FOXH1*, Forkhead box H1; *HAND2*, Heart and neural crest derivatives expressed 2; *JAG1*, Jagged-1; *FLNA*, Filamin-A; *KDR*, Kinase insert domain receptor (also known as VEGFR2); *NDRG4*, N-Myc downstream regulated 4; *SMARCC2*, SWI/SNF-related matrix-associated actin-dependent regulator of chromatin subfamily C member 2; *RYR1*, Ryanodine receptor 1; *ZFPM1*, Zinc finger protein multitype 1 (also known as Friend of GATA 1); *CAMTA2*, Calmodulin-binding transcription activator 2; *DLX6*, Distal-less homeobox 6; *PCM1*, Pericentriolar material 1.

In approximately 10% to 20% of patient, the specific genetic mutations responsible for the condition have yet to be pinpointed. A significant majority of NS cases are caused by *de novo* mutations, meaning the mutations arise spontaneously and are not inherited from parents. Genetic testing can provide valuable insights into the underlying genetic factors, facilitating better risk evaluation and guiding more effective patient management strategies. Although familiar forms of non-syndromic PVS have been described, the genetic etiology remains unknown [84].

The most common congenital heart abnormality, tetralogy of Fallot (TOF), is also linked to Pulmonary Valve Stenosis (PVS). TOF may be associated with subvalvular pulmonic stenosis, a defect obstructing the infundibular region that can be secondary to hypertrophy of the right ventricle. While nearly 20% of TOF cases are associated with identified diseases or chromosomal abnormalities, the other 80% of TOF cases are non-syndromic, meaning they have no known specific cause or underlying condition [77]. Non-syndromic TOF is a condition with a complex genetic basis. Both common and rare genetic variants seem to play a role in its development. Several genetic studies have been conducted, but only variants in the *NOTCH1* and *Fms-related tyrosine kinase 4* (*FLT4)* genes were discovered in approximately 7% of TOF cases. This finding suggests that these specific genes make significant contributions to the overall occurrence of TOF in the population. Other minority genes have been associated with non-syndromic TOF: *NK2 homeobox 5 (NKX2.5), GATA4, GATA5, GATA6, TBX1, T-box transcription factor 2 (TBX2), T-box transcription factor 5 (TBX5), Cbp/p300-interacting transactivator 2 (CITED2), Zinc finger protein multitype 2 (also known as Friend of GATA 2, ZFPM2/FOG2), Forkhead box C1 (FOXC1), Forkhead box C2 (FOXC2), Forkhead box H1 (FOXH1), heart and neural crest derivatives expressed 2 (HAND2), jagged-1 (JAG1), FLNA, Kinase insert domain receptor (KDR), N-Myc downstream regulated 4 (NDRG4), SWI/SNF-related matrix-associated actin-dependent regulator of chromatin subfamily C member 2 (SMARCC2), ryanodine receptor 1 (RYR1), zinc finger protein multitype 1 (ZFPM1), calmodulin-binding transcription activator 2 (CAMTA2), distal-less homeobox 6 (DLX6), pericentriolar material 1 (PCM1) * [78, 79]. Only a small number of individuals with non-syndromic TOF have been the subject of research, and our understanding of the key genetic factors responsible for this condition remains limited.

Williams syndrome (WS) is a relatively uncommon genetic disorder characterized by several features, and one of the frequent conditions associated with WS is congenital heart defects [80]. Supravalvular pulmonic stenosis is among the most prevalent, affecting around 45.1% of individuals with WS). The root cause of these cardiovascular issues lies in the deletion of the elastin gene on chromosome 7q11.23. This deletion results in insufficient or abnormal elastin deposition during cardiovascular development, leading to various cardiovascular abnormalities that manifest in individuals with WS [80].

Pulmonic regurgitation (PR) is relatively common in normal individuals and mainly caused by annulus dilatation secondary to pulmonary hypertension or dilatation of the pulmonary artery. Isolated congenital PR is rare and is more often a component of congenital disease as some forms of TOF [85].

## 6. Conclusions

There is undeniable evidence supporting a genetic component in both primary and secondary heart valve diseases. However, the genetic basis is intricate, given factors like incomplete penetrance, heterogeneity, and modifying factors. In cases where multigene panel testing does not provide a conclusive diagnosis for an individual displaying signs of familial valvulopathy, genomic testing options such as exome or genome sequencing may be considered as further diagnostic measures. These advanced genomic approaches offer a comprehensive analysis of the individual’s genetic makeup, aiding in identifying potential causative mutations or genetic factors contributing to the valvulopathy condition. It is important to note that the prevalence of certain valvulopathies, like mitral valve prolapse or bicuspid aortic valve, might be underrepresented in statistics due to their common and non-pathological occurrence in the general population.

Once a genetic basis is established in a case of heart valve disease, international guidelines strongly recommend screening first-degree relatives. However, systematic screening programs for this purpose are not consistently implemented, leading to occasional and potentially unequal screening practices.

In the last few years, researchers have focused not only on discovering new associated genes but also in the regulatory elements or post-transcriptional modifications. A new class of circulating biomarker called microRNA (miRNA) has emerged [86, 87]. miRNAs are a class of RNA molecules that do not code for proteins; instead, they govern the expression of genes at the post-transcriptional stage [86, 87]. Different studies focus on the identification of miRNAs in valvular heart diseases to open opportunities for new pharmacological targets or diagnostic screening [86, 87]. Although the genetic architecture of valvular heart disease is yet largely unknown, genetic testing in non-syndromic familial context, as well as in congenital disorders, is highly advisable for the purpose of discovering new genes involved in these disorders and the possibility to identify relatives at risk of the disease.
